# Long Term Amendment with Fresh and Composted Solid Olive Mill Waste on Olive Grove Affects Carbon Sequestration by Prunings, Fruits, and Soil

**DOI:** 10.3389/fpls.2016.02042

**Published:** 2017-01-09

**Authors:** Luca Regni, Luigi Nasini, Luana Ilarioni, Antonio Brunori, Luisa Massaccesi, Alberto Agnelli, Primo Proietti

**Affiliations:** Department of Agricultural, Food and Environmental Sciences, University of PerugiaPerugia, Italy

**Keywords:** *Olea europaea* L., organic waste, agro-ecosystem, C sequestration, soil fertility

## Abstract

The soil amendment with organic wastes represents a way to increase the soil fertility and the organic carbon (C) stored in the agro-ecosystems. Among the organic waste materials produced by agricultural and industrial activities, olive mill wastes derived from the olive oil extraction process may represent a suitable soil amendment. The aim of the study was to evaluate the effect of fresh (SOMW) or composted mixture of SOMW and shredded olive tree prunings (C-SOMW+P) on the vegetative and productive activities of olive trees, on the C stored in the tree non-permanent structures (prunings and fruits) and in the soil. The plots treated with SOMW or C-SOMW+P showed higher vegetative and productive activities than the untreated plots, and this was attributed to the higher total N and availability of P and K supplied by the amendments. Consequently, treatments increased the C sequestered in the tree non-permanent structures than in the control trees. However, no significant different effect between SOMW and C-SOMW+P treatments was found for the C stored in prunings and fruits, whereas it was evident a stronger influence of C-SOMW+P than SOMW on soil C sequestration. Indeed, about 50% the C supplied by the treatment with C-SOMW+P was sequestered in the olive grove system, with more than 90% of the sequestered C stored into the soil. The low amount of C sequestered in the soil following the addition of SOMW was attributed to its richness of moisture and easily degradable compounds that triggered the mineralization processes controlled by the soil microbial community. Although the 8 years of amendment produced a higher fruit yields than the control, no difference occurred between the characteristics and the oil content of the olive fruits. Only the total phenol content for the oil obtained from the SOMW-treated plots was significantly higher. The other considered fruit characteristics did not show significant differences.

## Introduction

As suggested by the UN convention panel on climate change, the ability to accurately assess the amount of C sequestered by soil and biomass in agro-ecosystems is becoming an increasingly important subject of worldwide interest [Intergovernmental Panel on Climate Change (IPCC), 2006; Teobaldelli et al., 2009; Hultnäs, 2011].

Indeed, the need for comprehensive strategies aimed at tackling the emission of greenhouse gases (GHG) has recently prompted research on the use of agricultural soils and perennial crops as potential sites for sequestration of organic C, although the long-term validity of this strategy is still to be assessed due to the variable behavior of immobilized C (Dumanski, 2004). Strategies based on changes in land use and agricultural management addressed in increasing the C stored in the tree biomass and into the soils have been reported to be potentially important to reduce atmospheric CO_2_ (Jarecki and Lal, 2003; Gómez et al., 2009). Among the agricultural managements, the addition to soil of organic wastes as amendments derived from various sources represents an efficient practice to increase soil organic C content in the agro-ecosystems (Mondini and Sequi, 2008; Sánchez-Monedero et al., 2008; Lozano-García et al., 2011; Bedbabis et al., 2015; Proietti et al., 2015; Russo et al., 2015). Furthermore, this strategy would bring other desirable benefits, such as enhancement of soil structure, increase soil fertility, and decrease soil erosion rate (Sánchez-Monedero et al., 2008). Such a “win–win” strategy would be particularly important in Mediterranean areas where the return of organic matter to the soil positively contributes to the mitigation of environmental problems, such as the severe degradation processes affecting some regions (Martínez-Mena et al., 2002). However, although many organic waste materials derived from agricultural activities and agro-industry could be used as effective soil amendments, they are still under-utilized and often considered as an environmental problem with high cost due to their disposal.

One of the most important and typical product of the agri-food industry of the Mediterranean region is the olive oil. The olive oil production annually generates, as by-product of extraction processes, a great amount of olive mill wastes (OMW) that could be considered a suitable soil amendment. Indeed, the high amount of organic C of OMW, made of both high-available water-soluble C and humic-like substances, and the abundance of N, P, and K makes this material an excellent soil conditioner with a good chemical fertilizing potential (Del Buono et al., 2011). Further, the recycling of OMW as soil amendments in the agricultural fields should avoid its disposal in landfills or burning in incineration plants that could bring, in addition, possible environmental concerns. The positive influence of OMW added to soil on the vegetative activity and yield of the olive trees has already been recognized by several authors (e.g., Altieri and Esposito, 2008; Giorgi et al., 2008; López-Piñeiro et al., 2008; Chartzoulakis et al., 2010; Camposeo and Vivaldi, 2011; Nasini et al., 2013; Proietti et al., 2015; Russo et al., 2015). Palese et al. (2013) demonstrated the potential of olive trees in sequestering C in an olive grove in Southern Italy, by calculating the biomass of the tree and the respective CO_2_ sequestration, and recent studies have shown that the application to soil of OMW-derived composts can improve long term C sequestration by the capacity of olive groves to store C in the tree biomass (Nasini et al., 2013).

Among the possible valorisation and/or recycling methods proposed for OMW (drying and solvent extraction of residual oil, biogas production, recovery of useful chemicals, composting, etc.), as reported in a previous study (Proietti et al., 2015), the direct land spreading on agricultural surfaces appears operationally simple and economically feasible. On the other hand, application of this waste material to the soil may have negative agronomical and environmental implications due to the acidic pH and the high content of potentially phytotoxic and anti-microbial compounds, such as phenols, tannins and fatty acids (Alburquerque et al., 2004). These negative aspects can be, however, partly overcome through the composting of OMW (Gigliotti et al., 2012; Nasini et al., 2016). Although, some authors reported the effect of the organic amendment derived from OMW on the olive grove productivity (Nasini et al., 2013; Palese et al., 2013; Proietti et al., 2015), and on soil physical-chemical characteristics (López-Piñeiro et al., 2011) and C sequestration (Sánchez-Monedero et al., 2008), as far as we know, no study has yet compared the impact of different forms of OMW on the productivity and on the capability to store carbon of the olive grove ecosystem. To fill this gap, the aim of the present work was to test the effects, in terms of productivity and C sequestration, of fresh and composted solid olive mill waste added to the soil of an olive grove for a long period. The effect of the fresh or composted amendments was evaluated during 8-year experiment on the vegetative and productive olive tree characteristics (canopy volume, leaf area dry mass (Leaf ADM), amount of prunings, leaf photosynthesis, and fruit yield, oil content and chemical characteristics) and on the C stored in the non-permanent tree structures (prunings and fruits) and in the soil of the olive grove. Prunings and fruit yields were chosen as indicators of the above ground biomass growth because any other measures of annual growth for above ground biomass would need destructive measurements on the studied trees, as described by Proietti et al. (2014). Specifically, we tested the following hypotheses: (i) the fresh OMW amendment increases vegetative activity and fruit yield of the olive trees; (ii) the fresh OMW amendment enhances a greater amount of C stored in non-permanent tree structure; (iii) the composted OMW amendment promotes a greater soil C storage than the fresh one; (iv) the use of amendments can affect the oil content and characteristics of the olive fruits.

## Materials and methods

### Olive grove characteristics

The study was conducted in a rain-feed olive grove, within a field station of the University of Perugia, located near Assisi (Perugia, Central Italy; 12°56′ E, 43°11′ N) at about 400 m a.s.l. The area has a continental climate. The mean annual air temperature is 13.5°C, with January as the coldest (−7°C) and July as the warmest (36°C) month, and a mean diurnal thermal range from 10 to 11°C. The mean annual precipitation is 810 mm, mostly concentrated in autumn, winter and spring, while in summer precipitation is very scarce.

The olive grove was established in 2000, after an 80-cm-deep breaking up, with olive *cv* Leccino at a planting distance of 5.5 × 5.5 m (331 plants ha^−1^). Leccino is a vigorous cultivar with a high and quite constant productivity; it is commonly cultivated in most olive growing areas and in particular in Central Italy. The soil of the olive grove, derived from calcareous marl, have a loam texture (41% sand, 34% silt, 25% clay) and was classified as a Typic Haploxerept (Soil Survey Staff, 2010). The soil is managed with natural and permanent green cover (two-three mowings are carried out each year and the mowed grasses left in place).

### Experimental design

The SOMW deriving from a three-phase oil extraction system, while C-SOMW+P was obtained by composting a mixture of olive pomace (SOMW), and shredded olive tree prunings (P) in the proportion 1:1 (v/v). Four blocks, each one consisting of 15 trees, were set up within the olive grove. From 2006 to 2013, during early spring, for each block five trees were amended with 150 kg per tree (about 50 tons ha^−1^) of SOMW, while other five trees were amended with 136 kg per tree (about 45 tons ha^−1^) of C-SOMW+P, and five trees were left as control. The different amount of fresh and composted amendment was added to the olive grove soil in order to supply the same amount of organic C. The spreading was done manually to 0.20 m from the trunk and affected the surface area under the projection of the canopy. During the thermophilic phase, a constant air flow (0.35 L h^−1^ kg^−1^ dry weight) was maintained. At the end of the thermophilic phase the material was placed in an open container without forced air until the curing phase was completed. The composting process was described in detail in a previous study (Proietti et al., 2015) and the chemical characteristics of SOMW and C-SOMW+P are reported in Table 1.

**Table 1:** **Mean chemical characteristics of the fresh and the composted SOMW (SOMW and C-SOMW+P, respectively) used as amendments**.

	**Moisture %**	**Conductivity mS cm^−1^**	**pH**	**TOC^a^, ^b^ g kg^−1^**	**TEC^a^, ^c^ g kg^−1^**	**Total N^a^ g kg^−1^**	**C:N ratio**	**P_2_O^a^ g kg^−1^**	**K_2_O^a^ g kg^−1^**
SOMW	44.1 (2.1) a	0.50 (0.01) a	6.60 (0.02) a	532.0 (3.9) a	103.0 (1.2) a	10.4 (0.2) a	51.4 (2.0) a	0.9 (0.01) a	5.6 (0.1) a
C-SOMW+P	28.3 (1.1) b	1.55 (0.02) b	7.35 (0.02) b	460.7 (3.3) b	164.9 (2.1) b	16.8 (0.4) b	27.42 (0.8) b	0.2 (0.01) b	7.6 (0.2) b

a *Values calculated on dry matter basis*.

b TOC: Total organic carbon;

c *TEC: Total extractable carbon. In each column, the mean values accompanied by different letters are significantly different for P < 0.05. Values in parentheses are standard errors of means*.

The spreading on the soil of SOMW and C-SOMW+P occurred in the early spring, and 1 week after the amendment both the treated and the control plots were fertilized with 0.3 kg of urea per tree. The organic amendment was not incorporated into the soil because it was cover cropped.

The fertilization was supplied to prevent temporary microbial immobilization of nitrogen during the degradation of the organic amendment, which could inhibit the vegetative activity of the olive trees. After about a week from the addition of urea, all plots received the standard nitrogen fertilization consisting of 150 kg ha^−1^ y^−1^ of urea. Even if the chemical characteristics of the organic materials used as amendments had a year-by-year variability due to differences in olive composition, the variations were small since each year the pomace was obtained from the same oil mill, in the same period and using olives harvested from the same olive grove.

### Vegetative activity and photosynthesis

The canopy volume was determined at the end of the summer before pruning.

Being the shape of the canopy similar to a cylinder, the volume of the canopy was calculated by,

$$\begin{array}{l} V=\pi \times r^{2}\times h \end{array}$$

where *h* is the height and *r* is the average radius of the canopy.

The fresh and dry weight of prunings obtained from each tree was recorded. The dry weight was determined by drying a representative sample for each tree at 105°C for 15 days.

Leaf net photosynthesis (Pn) was determined from 9:00 a.m. to 11:00 a.m. on cloudless days on 18 expanded current-season leaves and 18 1-year-old leaves, randomly sampled from well-lit canopy portions, at the beginning of May, June, September and October (corresponding to the phase of shoot growth, fruit set, fruit oil accumulation and veraison, respectively); the incoming photosynthetic photon flux density (PPFD) was about 1550, 1650, 1600, and 1500 μmol m^−2^ s^−1^, respectively. Pn was determined using a portable ADC LCA-3 gas exchange analyzer (Analytical Development Company Ltd., Hoddesdon, UK) and a Parkinson-type assimilation chamber. After the gas exchange measurements, the leaves were immediately transferred to the laboratory in a portable refrigerator for determination of the leaf area dry mass (Leaf ADM, obtained by leaf dry weight/leaf area); to this end, leaves were dried to constant weight in a forced air oven at 90°C.

### Fruit yield, fruit, and oil characteristics

At the beginning of October, detachment force, flesh firmness and pigmentation of about 50 olives per tree were determined following the well stated methodology (Camposeo et al., 2013). A “Carpo” hand dynamometer (“Effe.gi” dynamometer DT 05, Alfonsine-Ravenna, Italy) with 1.0 mm diameter tip was used and the “Jean pigmentation index,” ranging from 0 (100% intense green skin) to 7 (100% purple pulp and black skin).

Olive harvest was carried out at the end of October, and the fruit yield was recorded for each tree. The fruit dry matter was determined by drying a sample of 100 fruits randomly chosen for each tree at 105°C for 15 days. On 10 fruits per tree, the ratio between the fresh weight of the pulp and the fresh weight of the pit (P:N ratio) was calculated.

The fruit oil content was determined on four composite fruit samples (about 30 fruits each) by a NIR: Near Infra Red SpectraAlyzer (ZEUTEC Opto-elektronik, Rendsburg, Germany). Oil was extracted 1 day after the harvesting from two samples of about 2.0 kg per plot by using an artisanal mini oil mill as described by D'Amato et al. (2014). The oil chemical characteristics (acidity, peroxide number and total phenol content) were determined following the Official Methods of Analysis (Balestrieri et al., 1988).

### Determination of the C content of the tree non-permanent structures

The C content in non-permanent structures of the tree (leaves and wood of prunings, and fruits) was calculated by multiplying the amount of dry matter by 0.5, which represent, according Intergovernmental Panel on Climate Change (IPCC) (2003), the carbon fraction (CF) of the dry tree biomass (Hultnäs, 2011). The amount of C sequestered by the non-permanent structures in both treated and control plots was calculated, by multiplying the values on plant basis by 331 (number of plants per ha).

### Soil analyses

The soil of each amended and control plot, after removing the superficial green cover and amendment, was sampled by auger at 0–20 cm of depth, at 0.70 m from the trunk. Three samples within each plot were collected after 8 years of amendment. To determine the bulk density, three additional samples from each block were collected by cylindrical steel cores of known volume (100 cm^3^), oven dried at 105°C and weighted.

Before to be subjected to analysis, the soil samples were air-dried and sieving at 2 mm.

The soil pH was determined potentiometrically in water (solid:liquid ratio of 1:2.5). Total carbonates were determined by volumetric calcimeter method, whereas the active carbonates were assayed by treatment with 0.1 M ammonium oxalate and determination of the unreacted oxalate by titration with 0.1 M KMnO_4_ (Loeppert and Suarez, 1996).

Total N was determined by the Kjeldhal method, while the available P was determined according to Olsen method (Olsen et al., 1954). Exchangeable K was extracted by 1M NH_4_OAc at pH 7 and determined by flame emission spectroscopy (Sumner and Miller, 1996).

The total soil organic C (TOC) content was estimated by wet oxidation at 180°C for 30 min (Nelson and Sommers, 1996).

The stock of organic C accumulated in the considered thickness was calculated, taking into consideration C concentration, bulk density and thickness, by:

Stock of soil organic C (tons ha^−1^) = [C concentration (g kg^−1^) • bulk density (g cm^−3^) • thickness (m)] • 10.

### Statistical analysis

The data about the trees were determined for each tree every year of the treatment. The obtained data were averaged and the standard errors were calculated accordingly. The data about the soil were determined in triplicate for each plot at the end of the experiment.

After evaluation of the normal distribution of the data, the statistical analysis was performed by the one-way analysis of variance (ANOVA) and the means compared with Duncan's *post-hoc* test (*P* <0.05).

## Results

### Vegetative activity, fruit, and oil characteristics

The treatment with SOMW and C-SOMW+P did not affect the canopy volume of the olive trees and the Leaf ADM (Table 2). Conversely, both SOMW and C-SOMW+P treatments induced a higher amount of prunings than the control. Contrasting the two amendments, the amount of pruning dry weight was greater in the plots treated with C-SOMW+P. The leaf net photosynthesis was slightly higher for the trees of the plots treated with SOMW than for those treated with C-SOMW+P and control, which did not show differences between them (Table 2).

**Table 2:** **Canopy volume, pruning weight, leaf area dry mass (Leaf ADM) and leaf net photosynthesis (Pn) of the plants treated with fresh and composted SOMW (SOMW and C-SOMW+P, respectively) and the control (Assisi, central Italy)**.

	**Canopy volume**	**Prunings**		**Leaf ADM**	**Pn**
		**Fresh matter**	**Dry matter**		
	m^3^	kg per plant	kg per plant	mg cm^−2^	μmol CO_2_ m^−2^ s^−1^
SOMW	14.48 (0.94) b	22.31 (0.81) b	14.12 (0.79) b	25.25 (0.63) a	11.12 (0.39) b
C-SOMW+P	12.97 (1.03) a	23.60 (1.34) b	16.31 (1.01) c	25.28 (0.85) a	9.60 (0.73) a
Control	12.29 (0.71) a	19.56 (1.05) a	11.49 (0.47) a	25.20 (0.41) a	9.64 (0.97) a

*In each column, the mean values accompanied by different letters are significantly different for P <0.05. Values in parentheses are standard errors of means*.

The fruit yield was higher in the treated plots than in the control (Table 3), without statistically significant differences between the two treatments. Olives from the C-SOMW+P amended plots had a lower water content than those from the plots treated with fresh SOMW and from the control (39, 45, and 44%, respectively). No statistically significant difference was found for the oil content among the treated plots and the control. The other considered fruit characteristics (color, pulp firmness, detachment force, and pulp:pit ratio) did no show significant differences among the plots.

**Table 3:** **Yield, color, pulp firmness, detachment force, pulp:pit (P:N) ratio, and oil content of the fruits from the plants treated with fresh and composted SOMW (SOMW and C-SOMW+P, respectively) and the control (Assisi, central Italy)**.

	**Fruit yield**		**Colour**	**Pulp firmness**	**Detachment force**	**P:N ratio**	**Oil content**
	**Fresh matter kg per plant**	**Dry matter kg per plant**					
			**0–7**	***N***	***N***		**% dry weight**
SOMW	18.20 (1.32) b	10.04 (0.87) b	3.90 (0.12) a	4.03 (0.13) a	4.85 (0.21) a	2.53 (0.23) a	16.93 (0.36) a
C-SOMW+P	18.30 (1.55) b	11.22 (0.65) b	3.97 (0.17) a	3.99 (0.10) a	4.91 (0.18) a	2.45 (0.15) a	17.38 (0.21) a
Control	15.22 (1.04) a	8.52 (0.51) a	3.93 (0.09) a	4.00 (0.07) a	4.81 (0.13) a	2.36 (0.31) a	17.03 (0.47) a

*In each column, the mean values accompanied by different letters are significantly different for P <0.05. Values in parentheses are standard errors of means*.

Acidity and peroxide value of oil appeared to be not affected by the amendments (Table 4). Conversely, the amount of phenols was significantly influenced by the treatments. The highest amount of phenols was found in the oil produced from the olives of the trees treated with fresh SOMW, followed by the oil produced from the olives trees treated with C-SOMW+P, and by the oil from the control trees.

**Table 4:** **Chemical characteristics of oil obtained by the plants treated with fresh and composted SOMW (SOMW and C-SOMW+P, respectively) and the control (Assisi, central Italy)**.

	**Acidity %**	**Phenols mg kg^−1^**	**Peroxide value meq O_2_ kg^−1^**
SOMW	0.30 (0.15) a	509.75 (21.47) c	6.75 (0.02) a
C-SOMW+P	0.29 (0.10) a	442.95 (25.11) b	6.83 (0.10) a
Control	0.23 (0.09) a	361.78 (10.31) a	6.75 (0.04) a

*In each column, the mean values accompanied by different letters are significantly different for P <0.05. Values in parentheses are standard errors of means*.

### Soil characteristics

The amendment of the soil of the olive grove produced a reduction of the bulk density with respect to the control (Table 5), stronger in the plots treated with C-SOMW+P than in those treated with SOMW. Conversely, the pH values and the amount of total and active carbonates resulted unaffected by the amendments. The treatment with C-SOMW+P influenced the total N, the available P, and the exchangeable K contents. In fact, the C-SOMW+P treated plots had an amount of total N and available P 4- and 3-fold higher than SOMW-treated and control plots, respectively. Conversely, for total N and available P, the SOMW-treated and control plots did not show any significant difference among them. The content of exchangeable K was the highest in the soil of the plots treated with C-SOMW+P, followed by the SOMW-treated soil, and then by the control (Table 5).

**Table 5:** **Characteristics of the soils treated with fresh and composted SOMW (SOMW and C-SOMW+P, respectively) and the control (Assisi, central Italy)**.

	**Bulk density g cm^−3^**	**pH**	**Total carbonates %**	**Active carbonates %**	**Total N g kg^−1^**	**Available P mg kg^−1^**	**Exchangeable K mg kg^−1^**
SOMW	1.35 (0.04) b	8.1 (0.1) a	32.6 (1.1) a	12.5 (0.4) a	0.9 (0.2) a	17 (3.0) a	143 (17.0) b
C-SOMW+P	1.10 (0.1) c	8.1 (0.1) a	27.0 (0.7) a	11.4 (0.3) a	3.2 (0.7) b	57 (14.0) b	233 (39.0) c
Control	1.52 (0.1) a	8.2 (0.2) a	31.3 (0.6) a	12.8 (0.4) a	0.7 (0.1) a	20 (4.0) a	114 (11.0) a

*In each column, the mean values accompanied by different letters are significantly different for P <0.05. Values in parentheses are standard errors of means*.

### Organic C content in prunings, fruits, and soil

Considering the organic C concentration in the tree non-permanent structures (prunings and fruits), the two type of amendments had a positive effect with respect to the control, but did show no significant differences between them (Table 6). The C-SOMW+P treated plots had the greatest soil organic C concentration, whereas no difference was recorded between the soils of SOMW-treated plots and the control. Table 7 shows the balance between the organic C added with the two amendments and the C sequestered by soil and tree non-permanent structures (fruits and prunings) with respect to the control. After 8 years, although the same amount of C was supplied to the soil with the two treatments, the C-SOMW+P amended plots increased the soil C stock of 55.91 tons ha^−1^, whereas the SOMW treated plots increased their organic C stock of 2.65 tons ha^−1^. The long term addition of both SOMW and C-SOMW+P to the olive grove soil heightened the C sequestered by the tree non-permanent structures (Table 7). However, while for the C stored in the prunings the two amendments had a similar effect, the C fixed in the fruits in the 8-years experiment showed a slightly greater increase in the SOMW-treated plots than in those treated with C-SOMW+P (2.01 and 1.32 tons ha^−1^, respectively). From these data, it resulted that 8 years of amendment with SOMW and C-SOMW+P produced an increase of the C stock of the olive grove system (soil + tree non-permanent structures) of 8.13 and 60.49 tons ha^−1^, corresponding of the 6.84 and 50.87% of the organic C supplied, respectively.

**Table 6:** **Organic C content in the tree non-permanent structures and in the soil treated with the fresh and the composted SOMW (SOMW and C-SOMW+P, respectively) and the control (Assisi, central Italy)**.

	**Prunings**	**Fruits**	**Soil**
	**kg per plant**		**g kg^−1^**
SOMW	8.05 (0.27) b	5.51 (0.34) b	9.2 (2.2) a
C-SOMW+P	7.97 (0.31) b	5.25 (0.22) b	35.5 (3.5) c
Control	6.74 (0.68) a	4.75 (0.17) a	7.3 (1.9) a


*In each column, the mean values accompanied by different letters are significantly different for P <0.05. Values in parentheses are standard errors of means*.

**Table 7:** **Balance between the C added to soil during the 8 years of experimentation with SOMW and C-SOMW+P amendments (SOMW and C-SOMW+P, respectively) and C sequestered as soil organic C (SOC stock) and in tree non-permanent structures (C prunings, C fruit yield) with respect to the control (Assisi, central Italy)**.

	**C added in 8 years**	**SOC stock**	**C prunings**	**C olive yield**	**TotalC**	**% C sequestered by the olive-grove system**
	**Mg ha**^**−1**^					**%**
SOMW	118.96 a	2.65 a	3.47 a	2.01 a	8.13 a	6.84 a
C-SOMW+P	118.92 a	55.91 b	3.26 a	1.32 b	60.49 b	50.87 b

*In each column, the mean values accompanied by different letters are significantly different for P <0.05. The data of C sequestered by the non-permanent structures (prunings and fruits) were calculated, after the subtraction of the values of the control from those of the treated plots, by multiplying the values on plant basis by 331 (number of plants per ha)*.

## Discussion

The amendment with both SOMW and C-SOMW+P influenced the vegetative activity and yield of olive trees. Indeed, following the 8-years amendment, the amount of prunings (dry matter) increased with respect to the control of about 22.9 and 41.9% following the application of SOMW and C-SOMW+P, respectively, while the fruit yield increased, on dry matter basis, from 17.8 to 31.7%, although with no statistically significant effect between the two treatments. Similar olive yield increase was reported by López-Piñeiro et al. (2008) after 8 years of continued soil amendment with a fresh two-phase olive mill waste. A more general valuable effect of the olive mill waste application to soil on agricultural yields was reported by Brunetti et al. (2005), in which the higher wheat grain yield was attributed to the increase of the humic acid functional groups in the amended soils. The positive effect of the application of SOMW or C-SOMW+P on the vegetative activity and fruit yield can depend on the mineralization of the organic matter supplied with the amendments, and consequent release of nutrients, such as K, P, Ca, Mg, and Fe (Alburquerque et al., 2004; López-Piñeiro et al., 2011; Killi and Kavdir, 2013; Nasini et al., 2013; Fernández-Hernández et al., 2014).

Between the plots treated with the two amendments that differed for moisture content, pH, C/N ratio TEC, total N and P_2_O content (Table 1), a higher vegetative activity occurred in the C-SOMW+P-treated plots, as revealed by the higher amount of prunings produced. However, the net photosynthesis that was recorded in the SOMW-treated plots was slightly higher than that measured in the C-SOMW+P-treated and control plots. Further, these latter two did show no difference among them. The higher net photosynthesis induced by the SOMW may be attributed to a major N availability in the fresh SOMW, although its total N content was lower than that of the composted material (Table 1). The higher N availability was due to the lesser amount of N-bearing recalcitrant molecules in the SOMW than in the C-SOMW+P, which lost most of the inorganic N and the easily degradable organic molecules during the composting process (Gigliotti et al., 2012). The reduced N availability produced a marked decrease of the photosynthesis in many crops (Boussadia et al., 2010) because great part of the leaf N is in the photosynthetic apparatus (Makino and Osmond, 1991).

Although the 8 years of amendment produced a higher fruit yields than the control, no difference occurred between the characteristics and the oil content of the olive fruits from treated and untreated plots (Table 3). Our results did not agree with those of Fernández-Hernández et al. (2014), that found an increase of the olive oil content in the fruits from plots treated for 6 years with various olive mill waste-based composts with respect to the fruits from plots managed with inorganic fertilization. The SOMW and C-SOMW+P treatments did not strong affect the oil chemical characteristics. Indeed, only the total phenol content appeared to be influenced by the treatment, with the highest positive effect recorded for the oil obtained from the SOMW-treated plots, confirming that agronomical practices can affect the amount of the phenols in the olive oil (Cinquanta et al., 1997). Similar results has been previous reported by Fernández-Hernández et al. (2014), although the authors were not able to prove the direct influence of the application to soil of olive mill waste-based composts on the oil quality because of the lack of significant differences respect to the control. The higher total phenol content is attributable to the improved soil fertility and nutritional status of the trees due to the application of SOMW and C-SOMW+P (Fernández-Escobar et al., 2006; López-Piñeiro et al., 2011).

The increase of the phenol content in the olive oil from the treated plots is an interesting result because phenols have a positive effect on both the stability and the sensory and healthy characteristics of the oil.

Indeed, phenols have a positive wide range of biochemical and pharmaceutical effects, including anticarcinogenic, antiatherogenic, antimicrobial, and antioxidant activities (Visioli et al., 2004; D'Amato et al., 2014).

After 8 years of amendment, SOMW or C-SOMW+P had an impact on the soil physical and chemical properties. The significant lowering of the soil bulk density after the application of organic amendments, as observed in our experimentation, has been reported by several authors (Hemmat et al., 2010; Willekens et al., 2014), but, as far as we know, no reports are present in the literature that contrasts the effect of SOMW and C-SOMW+P on the bulk density. The lower soil bulk densities resulting by the application of C-SOMW+P could be attributed to the lesser density of the material due to the presence of about 50% wooden material (chipped prunings) in the composting mixture and its lower moisture. The lowering of the bulk density can be considered as an improvement of the soil physical fertility. Indeed, a lower bulk density implies a lesser soil compaction and a higher ability of the soil to function for structural support, water and solute movement, and aeration.

The C-SOMW+P had a greater efficiency in improving the soil fertility than SOMW, as indicated by the higher contents of total organic C, total N, available P, and exchangeable K found in the soil treated with the composted material. This effect, recognized by many authors (López-Piñeiro et al., 2008, 2011; Mechri et al., 2009; Chartzoulakis et al., 2010), was attributed mainly to the quality of the organic matter in this material that, at the end of the composting processes, resulted highly stabilized and recalcitrant to the mineralisation. This fact makes the composted material responsible for a slow and long-term release of nutrients (Murphy et al., 2007). The application of composted organic wastes, as well as favors the increase of organic matter in soil, it also promote the uptake or stocking of soil residual nutrients in such a way to prevent excess nutrient leaching into the groundwater. The fact that the nutrient release and their accumulation in soil are strongly linked to the cycling of the organic material applied to soil is supported by the scarce or not significant effect following the use of inorganic fertilizers (Nasini et al., 2013; Fernández-Hernández et al., 2014).

With regard to the potential C accumulation in the tree non-permanent structures it was evident that the improved nutritional status of the trees, that, in turn, was an effect of the higher nutrient availability due to the amendments, favored a higher vegetative activity and, consequently, a higher amount of C in the prunings and fruits of the treated plots with respect to the control. However, the application to soil of SOMW and C-SOMW+P had a similar impact on the C content in the tree non-permanent structures. Conversely, SOMW and C-SOMW+P had a different impact on the soil C stocks (Table 7). Indeed, while the SOMW-treated plots did not show any significant difference with the control, the C-SOMW+P-treated plots had an organic C content 5- and 4-fold higher than the control and the SOMW-treated plots, respectively. As hypothesized, the strong increase of the soil organic C content produced by the treatment with C-SOMW+P was attributed, other than to the presence of woody materials in the mixture, to the high degree of stabilization of the olive mill waste material acquired during the composting process. During this process, the mineralization of soluble compounds, coupled with polymerization and condensation reactions, leads to the formation of complex and highly recalcitrant organic molecules (Pichler and Kögel-Knabner, 2000; Said-Pullicino et al., 2007; Gigliotti et al., 2012). Further, the great loss of the soluble compounds, which occurs during the composting process of the olive mill wastes, generally produces a material rich in hydrophobic compounds, which enhance the chemical recalcitrance of the organic material (Piccolo et al., 1999; Brunetti et al., 2005; Doerr et al., 2005; Lorenz et al., 2007). The presence of recalcitrant compounds in the C-SOMW+P should contribute to the reduced mineralisation rate of C and its high residence time in soil (Gómez-Muñoz et al., 2012). In an 8-months incubation experiment where composted OMW (60–80% OMW, 10–20% olive leaf material, 10–40% poultry or sheep manure) was mixed with eight different soils, Gómez-Muñoz et al. (2012) found that less than 10% of the C was mineralised, independent of the soil characteristics.

The low amount of C sequestered in the SOMW-treated plots, where only 6.84% of the C added with the treatment remained in the olive grove system (Table 7), may be addressed to the higher content of moisture and easily degradable compounds that triggered the mineralization processes controlled by the soil microbial community (Bernal et al., 1998; Leifeld et al., 2002).

## Conclusions

The data obtained in this study showed that 8 years of application of SOMW and C-SOMW to the olive grove soil significantly increased the vegetative activity and fruit yield of olive trees, and phenols concentration in the oil. Further, the treatments reduced soil bulk density and increased the C sequestered in the tree non-permanent structures (prunings and fruits) and soil. The improved vegetative activity and fruit yield of olive trees can be attributed, especially for the C-SOMW, to a direct effect of the treatment on the amount of total N and availability of P and K in the soils. However, other factors indirectly associated to the addition of the organic amendments to olive grove soils, such as organic matter content and dynamic, water retention, and microbial biomass activity, could have influenced the improved vegetative activity and fruit yield of olive trees. From these results it appears that the recycling of the SOMW as a soil amendment, either composted or not, is a win-win strategy to transform a potential environmental threat associated to its disposal in a valuable resource and to improve the soil quality. However, no significant different effect of the SOMW and C-SOMW+P treatments occurred on C stored in pruning and fruits, whereas the C-SOMW+P showed a stronger influence than SOMW for soil C sequestration. Indeed, the high stability of C-SOMW+P made possible that about 50% the C supplied through the amendment is sequestered in the whole olive grove system, with more than 90% the sequestered C stored into the soil. The low amount of C sequestered in the SOMW-treated plots (only 6.84% of the C added with the treatment, with about 32% the sequestered C stored into the soil) was attributed to the higher microbial degradability of this material. Hence, although both SOMW and C-SOMW+P long term treatments positively affected the vegetative activity and fruit yield of the olive trees without changing the characteristics and the oil content of the olive fruits, the continuous application of composted olive mill wastes to the olive grove can represent an efficient strategy to increase both soil fertility and C sequestration efficiency.

## Author contributions

LR, LI, LM performed experimental work and data analysis. PP and AA designed the experiments. LN, LI made the table. LR, LI, LM, AA, and PP drafted the manuscript. All authors read and approved the final manuscript version.

### Conflict of interest statement

The authors declare that the research was conducted in the absence of any commercial or financial relationships that could be construed as a potential conflict of interest.
